# Can acute suicidality be predicted by Instagram data? Results from qualitative and quantitative language analyses

**DOI:** 10.1371/journal.pone.0220623

**Published:** 2019-09-10

**Authors:** Rebecca C. Brown, Eileen Bendig, Tin Fischer, A. David Goldwich, Harald Baumeister, Paul L. Plener

**Affiliations:** 1 University of Ulm, Department of Child and Adolescent Psychiatry and Psychotherapy, Ulm, Germany; 2 University of Ulm, Department of Clinical Psychology and Psychotherapy, Ulm; 3 Independent Contributor, Freelancing Data Journalist, Berlin, Germany; 4 Independent Contributor, Freelancing Software Developer, Berlin, Germany; 5 Medical University of Vienna, Department for Child and Adolescent Psychiatry, Vienna, Austria; University of Queensland, AUSTRALIA

## Abstract

**Background:**

Social media has become increasingly important for communication among young people. It is also often used to communicate suicidal ideation.

**Aims:**

To investigate the link between acute suicidality and language use as well as activity on Instagram.

**Method:**

A total of 52 participants, aged on average around 16 years, who had posted pictures of non-suicidal self-injury on Instagram, and reported a lifetime history of suicidal ideation, were interviewed using Instagram messenger. Of those participants, 45.5% reported suicidal ideation on the day of the interview (acute suicidal ideation). Qualitative text analysis (software ATLAS.ti 7) was used to investigate experiences with expressions of active suicidal thoughts on Instagram. Quantitative text analysis of language use in the interviews and directly on Instagram (in picture captions) was performed using the Linguistic Inquiry and Word Count software. Language markers in the interviews and in picture captions, as well as activity on Instagram were added to regression analyses, in order to investigate predictors for current suicidal ideation.

**Results:**

Most participants (80%) had come across expressions of active suicidal thoughts on Instagram and 25% had expressed active suicidal thoughts themselves. Participants with acute suicidal ideation used significantly more negative emotion words (Cohen’s d = 0.66, 95% CI: 0.088–1.232) and words expressing overall affect (Cohen’s d = 0.57, 95% CI: 0.001–1.138) in interviews. However, activity and language use on Instagram did not predict acute suicidality.

**Conclusions:**

While participants differed with regard to their use of language in interviews, differences in activity and language use on Instagram were not associated with acute suicidality. Other mechanisms of machine learning, like identifying picture content, might be more valuable.

Suicide is the second leading cause of death among adolescents and young adults according to the World Health Organization [1]. Especially suicidal ideation and suicide attempts reach high prevalence rates among adolescents. In a large study comprising 17 European countries, around one third of all adolescents reported lifetime suicidal ideation [2], with slightly higher rates in German school-based populations of 36.4–39.4% [3,4] and similar rates in first year college students [5]. A concerning 7–9% of German adolescents also report a lifetime history of suicide attempts [3,4,6].

Social media, of which Instagram is the most popular platform among adolescents [7,8], has become a fundamental channel for social interaction for adolescents [4]. Adolescents use social media as an essential communication strategy, disclosing information by generating, obtaining and sharing content [9,10]. The great use of social media among adolescents enables new approaches and perspectives to investigate suicide, as they provide big data sets of individual content [11–14], which is not influenced by laboratory settings and allows for exploration of everyday life communication. While several risk factors for suicidality have been described, prediction of suicide has not clearly progressed within the last decades, thus creating a need for new avenues, such as machine-learning based algorithms [15–17]. The analysis of social media behavior (e.g., posting pictures, connecting with others) and linguistic features of generated content (e.g., user posts) has been used to predict depression [18] and suicidal ideation [19].

Expression of acute suicidal thoughts is also a concerning phenomenon on social media, to which large providers like Facebook reacted by offering tools to help users who post online about their thoughts or plans of suicide (https://www.facebook.com/help/contact/305410456169423). In Chinese social media, Fu et al. [20] investigated responses to self-presented suicide attempts and expression of acute suicidal thoughts and found that written responses of other users often expressed caring or empathy, although cynical comments or shocked reactions were also common. However, little is known about how young people who express acute suicidal thoughts in social media perceive those reactions.

## Language use and suicidality

There are a number of cognitive and behavioral changes that have been described in users which are progressing towards verbalizing suicidal ideation. De Choudhury et al. [19] investigated posts and comments in mental health-related online forums. They designed a prediction framework that incorporates linguistic features (e.g., first person pronouns), linguistic structure (e.g. readability index) and interactional patterns (e.g., number of posts and comments). The framework was used to predict the individual risk of undergoing shifts from talking about general mental health issues to expressing suicidal ideation. Different linguistic features within this framework, like heightened self-attentional focus and poor interaction with the community characterized shifts from mere discussion of mental health issues to expressing suicidal ideation [19].

Current literature shows that it is possible to distinguish the level of concern among suicide related posts in social media using language-based classifiers [16,17,20,21]. This field of research is enabled by the availability of computer-based text analysis tools such as Linguistic Inquiry and Word Count (LIWC [22]). LIWC allows for a quantitative analysis of text with a focus on psychometric properties [23] and psychologically meaningful linguistic categories [24]. In the context of suicidal ideation, relevant linguistic markers include heightened self-attentional focus [18,25], a rise in negative emotion words [25], and changes in cognitive wording [26,27]. Additionally, authors reported poor readability (FRE; [28]) (i.e. the ease with which texts can be read/understood by the reader) [29–32] to be a marker for developing suicidal ideation [19].

## Aims of the current study

This is the first study to investigate language use on Instagram, one of the most prominent social media platforms among adolescents, concerning suicidality. The current study had two major aims: (1) to investigate the experience with expressions of acute suicidal thoughts on Instagram of young people using a qualitative approach and (2) to use LIWC as a quantitative approach to analyze differences in the language and Instagram activity of vulnerable young people (using qualitative interview data and captions on Instagram) with regard to their current suicidal ideation.

Regarding the first aim it was hypothesized that the majority of participants had come across expressions of acute suicidal thoughts on Instagram and that common reactions would include showing empathy or being shocked. We further hypothesized that expressions of acute suicidal thoughts would be met with an activation of a social help system on Instagram.

Regarding the second aim of the study, we hypothesized that in comparison to participants with past suicidal ideation only, participants with current suicidal ideation would use significantly more words related to a self-attentional focus (e.g. pronouns I, me, mine), negative emotions (e.g. fear, hate), their language would be defined by a high amount of cognitive words (e.g. confine, therefore) and a lower readability (FRE).

## Methods

### Data collection

Participants were identified from a larger data-set investigating the occurrence of non-suicidal self-injury (NSSI) on Instagram [33]. All pictures and user accounts associated with the 16 German hashtags most commonly related to featuring pictures of NSSI wounds (i.e. #ritzen (‘#cutting’) were downloaded at an hourly rate during four weeks in April 2016. For details on how those hashtags were identified see Brown et al. [33]. During data collection it was recorded how many followers users had, how many other users they were following, how many pictures they had posted and how many comments each picture had received. After those four weeks of Instagram data collection, a total of N = 100 randomly chosen users from this data-set were approached via Instagram messenger and asked if they were willing to participate in an interview-based study.

Interviews were conducted on Instagram messenger using chats, which allowed participants to stay anonymous. The interviews were semi-structured and consisted of 33 questions about the participants’ experiences with NSSI and suicidality on Instagram. Additionally, socio-demographic variables (i.e. gender, age) were assessed. Acute suicidality was assessed by the question: “Are you currently thinking about, or planning to, end your life?”. Lifetime suicidality was assessed by the question “Have you ever sincerely thought about ending your life?” and lifetime suicide attempts were assessed by the question “Have you ever tried to end your life?”.

### Ethics

Procedures contributing to this work comply with the ethical standards of the relevant national and institutional committees on human experimentation and with the Helsinki Declaration of 1975, as revised in 2008. All procedures involving human subjects were approved by the IRB of the Ulm University. Written informed consent via Instagram messenger was obtained from all subjects. Participants were informed about the purpose and risks of the study and about the use of their data for anonymous scientific publication via Instagram messenger. They agreed to those terms in written form via the messenger. All participants had to indicate to be over the age of 16. In case of acute suicidality they were provided with emergency help advice (nation-wide telephone numbers) and were offered to talk to a trained child and adolescent psychotherapist (RB) on the phone or via Instagram messenger. None of the participants made use of this option. Data was collected through the public Instagram API (https://www.instagram.com/developer) and was securely stored in an internal database. Access to the data is restricted to avoid personal identification of users and to comply with Instagram Terms of Use (https://www.instagram.com/about/legal/terms/) and API Terms (https://www.instagram.com/about/legal/terms/api/).

### Participants

Of the N = 100 users on Instagram who were initially approached, N = 64 agreed to participate in a qualitative interview regarding their experiences with suicidality and NSSI on Instagram, of which N = 59 completed the interview. Of those participants, N = 52 reported a lifetime history of suicidal ideation. Data of these 52 participants (of which n = 5 did not want to answer questions on socio-demographic variables, but completed the interview) are presented in the present paper.

### Qualitative data analyses

Semi-structured qualitative interviews were analyzed using the software ATLAS.ti 7. Two independent raters were thoroughly trained. Taking an example of three interviews, and paraphrasing them, categories from those paraphrased responses were generated in order to facilitate standardized ratings. Raters were trained continuously until the first five rated interviews showed very good inter-rater-reliability of a minimum of kappa = .80. The rest of the interviews were rated under ongoing supervision. Answers to the following questions were analyzed in the current study: “Have you ever announced a future suicide attempt on Instagram?” and “What were reactions of other users to this announcement?” as well as “Have you ever come across someone announcing suicide on Instagram?” and “What were reactions to this announcement?”. Inter-rater reliability ranged from substantial agreement (kappa = .61) for the category “Worried reactions to suicide announcements online” to perfect agreement (kappa = 1.0) for the categories “My account was reported to Instagram after my suicide announcement” and “Other users reacted shocked to my suicide announcement”. Whenever there was a disagreement between two ratings, an agreement was found between both raters and one of the authors of the paper (RB).

### Quantitative data analyses

Qualitative interview data and captions on Instagram were analyzed using the Linguistic Inquiry and Word Count (LIWC) software. Instagram data from N = 52 users, who answered the question about acute suicidality (N = 25 participants reported acute suicidality, N = 27 no acute suicidality, but suicidal ideation in the past) were analyzed. Features measuring linguistic style were extracted.

For word count and linguistic analysis, the German dictionary of the LIWC was used [34]. The LIWC is a computer-based text analysis software tool whose algorithms count words according to pre-defined criteria word categories [34]. The resulting output file from this analysis contains information in percent (frequency of specific words in relation to the total number of words).

Furthermore, we used the Flesch-Reading-Ease index (FRE; [28,30]) to calculate the ease with which one can read or understand responses given by adolescents. The FRE is normalized to values between 0 and 100 with higher values indicating high reading ease (0–30 very low reading ease, comprehensible for academics; 30–50 low, 50–60 medium, 60–70 well understandable texts; 70–80 medium understandable, 80–90 easy and 90–100 very high reading ease, comprehensible for eleven-year old pupils) [35]. The index is calculated using the average sentence length (ASL) and the average number of syllables per word (ASW) [28]. Based on previous research on language use and suicidality, we chose the following specific variables from the LIWC and the automated readability index for analyses:

1. Category of negative emotion words (e.g., sad, angry, hatred)
2. Category of overall affect (emotion expression)
3. Category of cognitive words (e.g., because, understand, but)
4. Category of first person pronouns (I, my, mine)
5. Automated readability index (*FRE*_*german*_ = 180 − ASL − (58,5 * ASW))

As a further feature of the quantitative analysis, activity on Instagram (number of followers, number of following others, number of pictures posted, average number of comments per picture) within the past month was taken into account.

### Statistical analyses

Statistical analyses were performed with R [36]. Differences between participants with acute vs. non-acute suicidality were calculated using *t*-tests. Effect sizes (Cohen’s *d*) were calculated for significant differences. Based on previous research [19], logistic regression analysis was calculated with acute vs. non-acute suicidality as dependent variable and linguistic features (expression of negative affect, pronoun, cognitive mechanism, emotion expression, readability index) as well as activity on Instagram (number of followers on Instagram, number of users on Instagram they were following, number of pictures posted within the past month, or number of comments other users posted in response to those pictures on average per picture) as independent variables.

## Results

Of the N = 47 participants providing information on socio-demographic details, N = 41 (87%) stated to be female. Participants were on average 16.6 years old (SD = 0.96, min = 16, max = 20), with a median age of 16 (interquartile range = 16 to 17 years). Most participants attended school (N = 38, 80.9%), followed by 14.9% (N = 7) who went to university or were in professional training, and 4.3% (N = 2) who were currently unemployed.

### Qualitative data

All participants reported a lifetime-history of suicidal ideation and NSSI and 45.5% (N = 25) reported suicidal ideation on the day of the interview. Around half of all participants (53.8%, N = 28) reported a lifetime history of suicide attempts, and 23.5% reported a suicide attempt within the past month (N = 12).

Of all participants, 13 (25%) reported to have announced a planned suicide on Instagram. Asked about the reaction of other users to their announcement, the following themes emerged: “People offered help” (N = 6), “People tried to talk me out of it” (N = 8), “My account was reported to Instagram (N = 2), “People suggested a joined suicide” (N = 2), “People were shocked, sad, and devastated” (N = 1), “People encouraged me to commit suicide” (N = 1). Four participants reported ‘actual’ action by other users in reaction to their announcement (calling the police, telling parents), while all other perceived reactions remained purely online.

The majority (80.8%, N = 42) of all participants reported to have come across a expression of acute suicidal thoughts online. The following themes emerged when asking about reaction to those suicide announcements: “people were worried” (N = 30), “people showed empathy” (N = 5), “people encouraged the person to commit suicide” (N = 5), “people were helpless” (N = 2), “people reported the user to Instagram” (N = 2), “people expressed to not understand the person” (N = 2), “people identified with the person” (N = 1).

### Quantitative results

Participants with suicidal ideation on the day of the interview (‘acute suicidality’, AS) were compared to participants with past, but without current suicidal ideation (‘non-acute suicidality’, NAS).

Gender, age, occupational status, or lifetime attempted suicide were not associated with acute suicidality, and neither was activity on Instagram in the past four weeks. That is, number of followers on Instagram, number of users on Instagram they were following, number of pictures posted within the past month, or number of comments other users posted in response to those pictures on average per picture did not differ between the two groups (see Table 1).

**Table 1:** Demographic characteristics and Instagram activity within the past four weeks.

	Acute suicidality N = 25	Non-acute suicidality N = 27		
	Mdn (IQR)	Mdn (IQR)	Mann-Whitney-U-Test (p)	Z
Age	16.0 (16.0–17.0)	16.5 (16.0–17.0)	255.0 (0.2)	1.25
	N (Pct.)	N (Pct.)	Chi^2^ (p)	df
Gender			0.51 (.48)	1
female	21 (91.3%)	20 (83.3%)		
male	2 (8.7%)	4 (16.7%)		
Occupation			3.07 (.22)	
High-school student	20 (83.3%)	20 (76.9%)		
University / professional training	4 (16.7%)	3 (11.5%)		
Unemployed	0	3 (11.5%)		
Lifetime suicide attempt	16 (64.0%)	12 (40.0%)	3.14 (.08)	1
Characteristics on Instagram	Mdn (IQR)	Mdn (IQR)	Mann-Whitney-U-Test (p)	Z
Number of followers	123 (7.0–249.0)	55.0 (5.0–217.0)	255.0 (0.3)	1.08
Number of following others	60.0 (14.0–122.0)	36.0 (11.0–97.0)	279.0 (0.5)	0.61
Number of pictures posted	13.0 (3.0–45.5)	7.0 (3.0–13.0)	264.5 (0.3)	1.09
Average number of comments per picture	2.0 (0.4–4.7)	1.0 (0.4–2.65)	252.5 (0.2)	1.32

*Note*: N = number of participants, Pct. = Percent, Mdn = median, IQR = interquartile range, p = level of significance, Z = Z-Score, df = degrees of freedom, Chi^2^ = Chi^2^ value

Language analyses were calculated separately for language use in interviews and language use in captions on Instagram, respectively.

### Results concerning language in interviews

On average, participants in the AS group used significantly more negative emotion words (*M* = 1.95, *SD* = .52) than participants in the NAS group (*M* = 1.57, *SD* = .63). For psychological processes, participants in the AS group used significantly more words reflective of emotion expression (*M* = 5.71, *SD* = .98), than participants in the NAS group (*M* = 5.13, *SD* = 1.05). All other differences were not significant (see Table 2).

**Table 2:** Language analyses of interviews and captions.

	Acute suicidality	Non-acute suicidality			
Language in interviews					
	M (SD)	M (SD)	T (p)	df	Cohens´d (CI)
Pronoun	12.45 (2.27)	11.90 (1.75)	1.28 (.34)	45	0.27 (-0.31–0.85)
Emotion expression	5.71 (0.98)	5.13 (1.05)	2.06 (.04)	50	0.57 (0.00–1.14)
Negative emotions	1.95 (0.52)	1.57 (0.63)	2.39 (.02)	49	0.66 (0.09–1.23)
Cognitive mechanism	13.29 (1.59)	12.60 (1.84)	1.46 (.15)	50	0.40 (-0.15–0.96)
Readability (FRE)	64.64 (16.67)	60.16 (14.68)	1.03 (.31)	48	0.29 (-0.28–0.85)
Language in captions					
Pronoun	5.06 (3.21)	4.01 (2.74)	1.26 (.21)	47	0.35 (-0.22–0.92)
Emotion expression	8.47 (5.04)	9.18 (5.60)	0.48 (.63)	50	-0.13 (-0.68–0.42)
Negative emotions	6.95 (4.69)	7.85 (5.66)	-0.63 (.53)	50	-0.17 (-0.72–0.38)
Cognitive mechanism	4.03 (2.36)	3.75 (2.27)	0.44 (.66)	50	0.12 (-0.43–0.67)

*Note*: N = number of participants, M = Mean, SD = standard deviation, T = t-value, p = level of significance, df = degrees of freedom, CI = 95% Confidence interval

In a step-wise logistic regression analysis combining language use in interviews and Instagram activity (except language in captions), only expression of negative emotion in the interviews was significantly associated with acute suicidality (Regression-coefficient B = 1.19, p = .029, OR = 3.28 (95% CI: 1.10 to 9.77), while we did not find associations with any of the other variables (pronoun, cognitive mechanism, emotion expression, number of followers, number of users following, pictures posted, average of comments per picture) (see Table 3).

**Table 3:** Results of the binary logistic regression.

Model	B	SE (B)	AIC	p
Step 1			70.45	
Constant	2.17	1.0		.031
Negative Emotion	1.19	0.54		.028
Step 2			71.71	
Constant	3.31	1.71		.052
Negative Emotion	0.89	0.64		.16
Emotion Expression	0.30	0.35		.39

*Note*: B = Regression coefficient, SE (B) = Standard error of the regression coefficient, AIC = Akaike information criterion, p = level of significance

The final model with negative emotion as associated variable was applied to the data to calculate the odds for AS of each individual. Based on a cut-off for the calculated odds, participants were defined as AS or NAS. Predicted AS was compared to reported AS by participants. Accuracy, sensitivity and specificity varied depending on cut-off: Maximal accuracy of 69.23% was achieved at a cut-off of 0.7 (sensitivity = 84%, specificity = 56.56%) (see Supporting Information, S1 Table, S1 Fig).

### Results concerning language in captions

Participants in the acute suicidality group did not differ from participants in the non-acute suicidality group regarding their use of language in captions on Instagram in the four weeks prior to the interview (for details see Table 3). Prediction models with pronouns, negative emotion, cognitive mechanism, and emotion expression as factors were applied to language in the captions. No significant predictors could be found (all p>.05) (Table 3).

## Discussion

In this sample of young Instagram users with a lifetime history of suicidal ideation and NSSI, half of all participants had attempted suicide at least once and half of them were expressing acute suicidality on the day of the interview. These characteristics constitute this group of Instagram users as a very vulnerable at-risk group for suicidality. A quarter of all participants in this study reported to have expressed acute suicidal thoughts on Instagram. Reactions by other Instagram users indicated empathy, the activation of a social help system by other users offering help, trying to talk them out of it, or indicating sadness or shock. However, only in around a third of the cases, action was taken by informing the police or parents. Additionally, around 80% of all participants reported to have come across a suicide announcement online. Again, reactions of other Instagram users were mainly trying to offer help, by showing empathy, being worried, or reporting the user to Instagram. However, in both cases (either actively posting online about their thoughts or plans of suicide or coming across a suicide announcement online), a small percentage of participants reported incidents of other users encouraging the person to commit suicide or suggesting a joint suicide. No actions by Instagram (i.e. immediate deletion of the comment) were reported by the interviewed participants. These results are in line with former studies investigating responses to expressions of acute suicidal thoughts in social media [20]. These should comprise the discussion of ethical questions and practical implications for future suicide prevention in social media [13] which could result in stricter legal requirements for social media providers regarding comments in the context of suicidality.

The detection of suicide risk through social media might be an opportunity for accurate and timely identification of acute suicidality [9], e.g. by using language variables for predictive analytics [19,37]. In this line, the second aim of this study was to investigate whether participants with current suicidal ideation would differ in their language use as well as in their Instagram activity from participants with non-acute suicidality in this German speaking sample. In order to control for situational biases of language used in captions on Instagram, data of qualitative interviews was also used to test for differences in language use. Overall, Instagram activity did not distinguish between participants with acute versus non-acute suicidality (neither language use in captions nor number of followers, pictures posted, comments, etc.). This is somewhat contrary to a study by De Choudhury et al. [19], who found language use and some activity markers (e.g. length of comments posted) in mental health forums on Reddit to be predictable of suicidal expressions. However, this might be due to the different nature of Reddit (where the main content is shared in language based discussion forums) and Instagram (where the main content is shared through pictures). According to our results, in this highly vulnerable group of participants posting pictures with NSSI on Instagram, automated linguistic analyses of data shared on Instagram might not be feasible to detect persons at risk. Approaches that apply machine learning tools to Instagram photos might be more promising in this context [38].

In language data obtained from qualitative interviews, significant differences between participants with AS vs. NAS could be found for negative emotion words and emotion expression with medium effect sizes. A binary logistic regression model revealed that for each unit (percentage) increase of negative emotion words, the odds for acute suicidality increased about 3 times. Differences regarding self-attentional focus and cognitive words were non-significant, but indicated the same direction as previous studies. Considering the homogeneity of the participants in this study regarding past NSSI and suicidal ideation (100% reported suicidal ideation and had posted pictures of NSSI on Instagram), and the rather small sample size, it is quite remarkable that effects found in previous studies seem to be robust in the current study and point towards a rather high validity of using interview data as compared to using captions in social media for language analyses. Even though interviews were conducted on Instagram messenger, language in those interviews was quite coherent (e.g. full sentences), while data in Instagram captions was usually quite fractured (e.g. changing between English and German in the middle of a sentence, heavy use of Emojis, use of incomplete sentences or single words). Interestingly, the overall use of emotional words in both groups seemed to be twice as high in captions as compared to interviews, while participants used around twice as many pronouns and cognitive words in interviews. This may have also biased linguistic calculations regarding captions. However, analysis of Instagram data should possibly include machine learning algorithms trained on picture- rather than on language data [38].

The calculated accuracy of our model indicates that in 69 percent of cases, participants could be correctly assigned to acute suicidality and non-acute suicidality based on language data in interviews (Meaningful prediction of acute suicidality based on captions was not possible (all p>.05). Although this prediction model is depending on the present sample, it achieved highly similar accuracy and slightly higher specificity and sensitivity as a supervised machine learning model of Nobles et al. [16], which correctly assigned participants in 70 percent of cases to depression and suicidality, with a sensitivity of 81% and a specificity of 56%. Further studies could use the prediction model (see supplementary material) to test the predictive value in other samples. Machine learning algorithms trained on larger datasets and incorporating additional information, like e.g. acoustic features [39,40] might be a fruitful approach to further investigate this finding.

Methodological limitations are related to the exploratory character of this study and the small sample size. Therefore, results of this study have to be interpreted with caution and cannot be generalized to other populations. Additionally, there was no “never suicidal/NSSI” control group to which we could have compared the average word use (e.g. Instagram users who had not posted pictures of NSSI). The LIWC might be a well-validated instrument to reveal information pertaining to psychological aspects [23], but a major problem of the software is that there are no standard values available to compare data to the general population. Furthermore, data of participants was completely anonymous, as interviews were conducted on Instagram messenger. Therefore, socio-demographic data cannot be validated. There may have also been a self-selection bias of mainly female adolescents choosing to participate in the current study.

Social media platforms are increasingly integrating mechanisms to detect suicidal posts and have started to implement automated help suggestions. With recent advances in machine learning and data mining, massive amounts of data can be used for predictive models, opening up new avenues for detection and prevention of suicidal behavior [15]. For example, a recent study using Twitter data showed that users posting in ‘suicidal networks’ seem to be much more closely connected than other Twitter users. Those network analyses could be interesting for future investigations of Instagram data. However, ethical challenges when analyzing mental health data of social media users have to be taken into account [41], and data generated by at-risk individuals might not always be accurately pointing towards a risk for suicidality. Furthermore, our findings point to the fact that language based machine-learning algorithms might be limited in their ability to detect suicidality among users when used in mostly picture based social media, like Instagram. Other mechanisms of machine learning, which are also capable identifying picture content might be more helpful. According to the reports of participants of the current study, Instagram did not take active and effective measures to prevent suicide or possible contagion effects of suicidal ideation. Overall, social media providers need to be aware of at-risk users within their networks and need to take action when necessary. Mental health care providers should be aware of their patients’ social media use, address it, and discuss benefits and risks with their patients. Reading active suicidal thoughts online might be disturbing and should be addressed accordingly. Within the network of participants posting pictures of NSSI, universal preventative measures could be implemented, as a large number of those young people seem to be at risk for suicide. Furthermore, there seems to be a potential for using social media in a protective way, as it has recently been shown that fictional peer comments, can have an impact to positively change attitudes towards recovery from NSSI [42].

## Supporting information

S1 Fig Trends in accuracy, sensitivity and specificty. (DOCX)

S1 Table Trends in accuracy, sensitivity and specificity. (DOCX)
