# Supplementary figures and images for: Can acute suicidality be predicted by Instagram data? Results from qualitative and quantitative language analyses
Source: PLoS One. 2019 Sep 10;14(9):e0220623. doi: 10.1371/journal.pone.0220623 (PMC6736249; doi:10.1371/journal.pone.0220623)

Figure 1A. Trends in accuracy, sensitivity and specificty.


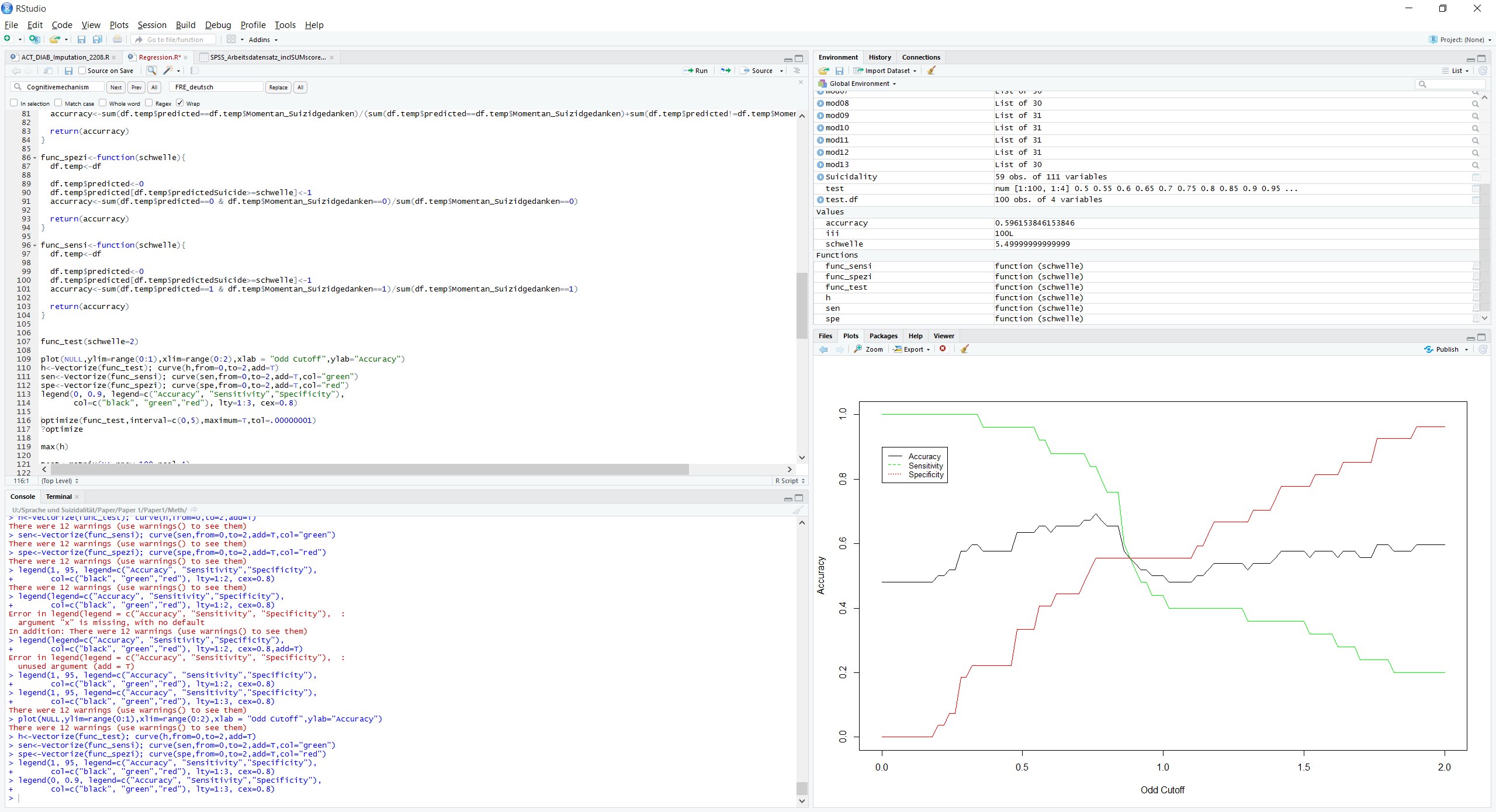

Supplement: S1 Fig — (DOCX) [file pone.0220623.s001.docx]
