# Supplementary material for: Can acute suicidality be predicted by Instagram data? Results from qualitative and quantitative language analyses
Source: PLoS One. 2019 Sep 10;14(9):e0220623. doi: 10.1371/journal.pone.0220623 (PMC6736249; doi:10.1371/journal.pone.0220623)
Supplement: S1 Table — (DOCX) [file pone.0220623.s002.docx]

Table 1A: Trends in accuracy, sensitivity and specificty.

|  | Treshold for Odds | Accuracy | Sensitivity | Specificity |
| --- | --- | --- | --- | --- |
| 1 | 0.5 | 0.634615385 | 0.92 | 0.37037037 |
| 2 | 0.55 | 0.634615385 | 0.88 | 0.407407407 |
| 3 | 0.6 | 0.653846154 | 0.88 | 0.444444444 |
| 4 | 0.65 | 0.653846154 | 0.88 | 0.444444444 |
| 5 | 0.7 | 0.692307692 | 0.84 | 0.555555556 |
| 6 | 0.75 | 0.653846154 | 0.76 | 0.555555556 |
| 7 | 0.8 | 0.615384615 | 0.68 | 0.555555556 |
| 8 | 0.85 | 0.538461538 | 0.52 | 0.555555556 |
| 9 | 0.9 | 0.519230769 | 0.48 | 0.555555556 |
| 10 | 0.95 | 0.5 | 0.44 | 0.555555556 |
| 11 | 1 | 0.480769231 | 0.4 | 0.555555556 |
